# Improving the quality of paediatric malaria diagnosis and treatment by rural providers in Myanmar: an evaluation of a training and support intervention

**DOI:** 10.1186/s12936-015-0923-9

**Published:** 2015-10-09

**Authors:** Tin Aung, Kim Longfield, Nyo Me Aye, Aung Kyaw San, Thea S. Sutton, Dominic Montagu, PHSi/UCSF Global Health Group

**Affiliations:** Research Department, Population Services International-Myanmar, Yangon, Myanmar; Strategic Research and Evaluation, Population Services International, Washington, DC, USA; UCSF Global Health Sciences, San Francisco, CA USA; Private Health Sector Initiative (PSHi), UCSF Global Health Group, 550 16th Street, 3rd Floor, Box 1224, San Francisco, CA 94158 USA

**Keywords:** Social marketing, Social franchising, Quality, Private providers, Rural, Myanmar, Malaria, Community health workers, Volunteer health workers

## Abstract

**Background:**

This study evaluates the effectiveness of a training programme for improving the diagnostic and treatment quality of the most complex service offered by Sun Primary Health (SPH) providers, paediatric malaria. The study further assesses whether any quality improvements were sustained over the following 12 months.

**Methods:**

The study took place in 13 townships in central Myanmar between January 2011 and October 2012. A total of 251 community health workers were recruited and trained in the provision of paediatric and adult malaria diagnosis and treatment; 197 were surveyed in all three rounds: baseline, 6 and 12 months. Townships were selected based on a lack of alterative sources of medical care, averaging 20 km from government or private professional health care treatment facilities. Seventy percent of recruits were assistant nurse midwives or had other basic health training; the rest had no health training experience. Recruits were evaluated on their ability to properly diagnosis and treat a simulated 5-year-old patient using a previously validated method known as Observed Simulated Patient. A trained observer scored SPH providers on a scale of 1–100, based on WHO and Myanmar MOH established best practices. During a pilot test, 20 established private physicians operating in malaria-endemic areas of Myanmar scored an average of 70/100.

**Results:**

Average quality scores of newly recruited SPH providers prior to training (baseline) were 12/100. Six months after training, average quality scores were 48/100. This increase was statistically significant (p < 0.001). At 12 months after training, providers were retested and average quality scores were 45/100 (R3–R1, p < 0.001).

**Conclusion:**

The SPH training programme was able to improve the quality of paediatric malaria care significantly, and to maintain that improvement over time. Quality of care remains lower than that of trained physicians; however, SPH providers operate in rural areas where no trained physicians operate. More research is needed to establish acceptable and achievable levels of quality for community health workers in rural communities, especially when there are no other care options.

## Background

With a population of approximately 51.4 million people, Myanmar is one of the poorest countries in Asia and is classified by the United Nations as one of the least developed countries in the world [1]. Health indicators are some of the worst in the world: life expectancy is only 65 years, and rates of child and maternal mortality are high. In addition, there are high rates of child malnutrition and stunting, diarrhoeal diseases and pneumonia are common, and Myanmar has more than half of all malaria-related deaths in southeast Asia [2–5].

Government spending on healthcare in Myanmar is minimal. Between 2007 and 2011, expenditures were only US$17 per person per year, the lowest in Asia and far below the $60 per person benchmark recommended by the World Health Organization (WHO) for low-income countries hoping to reach the Millennium Development Goals by 2015. The health system challenges are large and ongoing [8]. Major new investments are beginning, but in the 2011/12 fiscal year, the government spent just 1.3 % of its overall budget (US$110 million) on healthcare. This lack of government support means that more than 90 % of healthcare expenditures in Myanmar are out of pocket [6, 7].

National health service coverage is low and access to healthcare is limited. General practitioners (GPs), voluntary organizations, and international non-governmental organizations (INGOs) provide a significant fraction of all healthcare in Myanmar [10]. In 2010 there were 24,536 total doctors: 9728 in the public sector and the remainder in the private sector. The number of physicians per 1000 people was 0.5 in 2008, constituting a shortage of health workers according to WHO norms [10, 11]. The shortage is worst in rural areas, where 66 % of Myanmar’s population is concentrated [9]. In addition to health worker deficits, travel restrictions within the country often prevent reliable access to healthcare when it is available; travel restrictions are most common in rural areas [5].

As is the case in most low- and middle-income countries (LMICs), in the absence of sufficient public health services, 80 % of the Myanmar people seek their healthcare from the private sector [12–14]. There is a perception that the private sector can provide a more reliable drug supply, better responsiveness, and a more client-centered focus [13, 15–19].

A common method for dealing with healthcare worker shortages and improving access to healthcare services, especially in rural and poor areas, is “task shifting” [19, 20]. Task shifting is the simplification and delegation of health tasks from medically trained doctors to other providers [19, 21]. In some Myanmar villages, the Department of Health has trained community health workers (CHW) and auxiliary midwives to deliver care in the absence of primary healthcare services. However, these CHW positions are non-salaried and have a high rate of attrition [10].

Recent systematic reviews for LMICs have highlighted the need to standardize and ensure the levels of quality offered by both public and private providers, especially in rural settings [15, 22, 23]. By increasing the quality of care, both sectors can improve cure rates and decrease unnecessary treatment, reducing out-of-pocket expenditures on healthcare, especially among the poor [15].

Since the 1990s, clinical social franchising has become an increasingly popular method for delivering healthcare to the poor. Social franchising aims to strengthen business practices through economies of scale. The franchisor, typically an international NGO with an in-country office, recruits and supports network members through branding private clinics and purchasing drugs in bulk at wholesale prices. The primary advantage of this model is the potential for fast, low-risk expansion via pre-existing clinics and pharmacies, backed by a recognized brand with well-established attributes desired by consumers [24, 25].

Population Services International (PSI) is a US-based NGO that opened its first office in Myanmar in 1995. In 2001, PSI/Myanmar (PSI/M) started its social franchising programme with the Sun Quality Health network, and focused on private providers in urban and peri-urban settings. In 2008, it established the Sun Primary Health (SPH) network to increase rural coverage of priority health services: malaria prevention and treatment, reproductive health (RH) services, pneumonia diagnosis and referrals, diarrhoea prevention and treatment, and referrals to treatment facilities for tuberculosis (TB). SPH providers are typically auxiliary midwives, retired nurses, teachers, farmers, or housewives residing in the rural areas they serve. In 2011 PSI initiated a major expansion of the SPH Network.

This study evaluated the effectiveness of a training programme for improving the diagnostic and treatment quality of paediatric malaria by SPH providers: the service area with the most complex training components. The study further assessed whether any quality improvements were sustained over the following 12 months.

## Methods

**Fig. 1:**
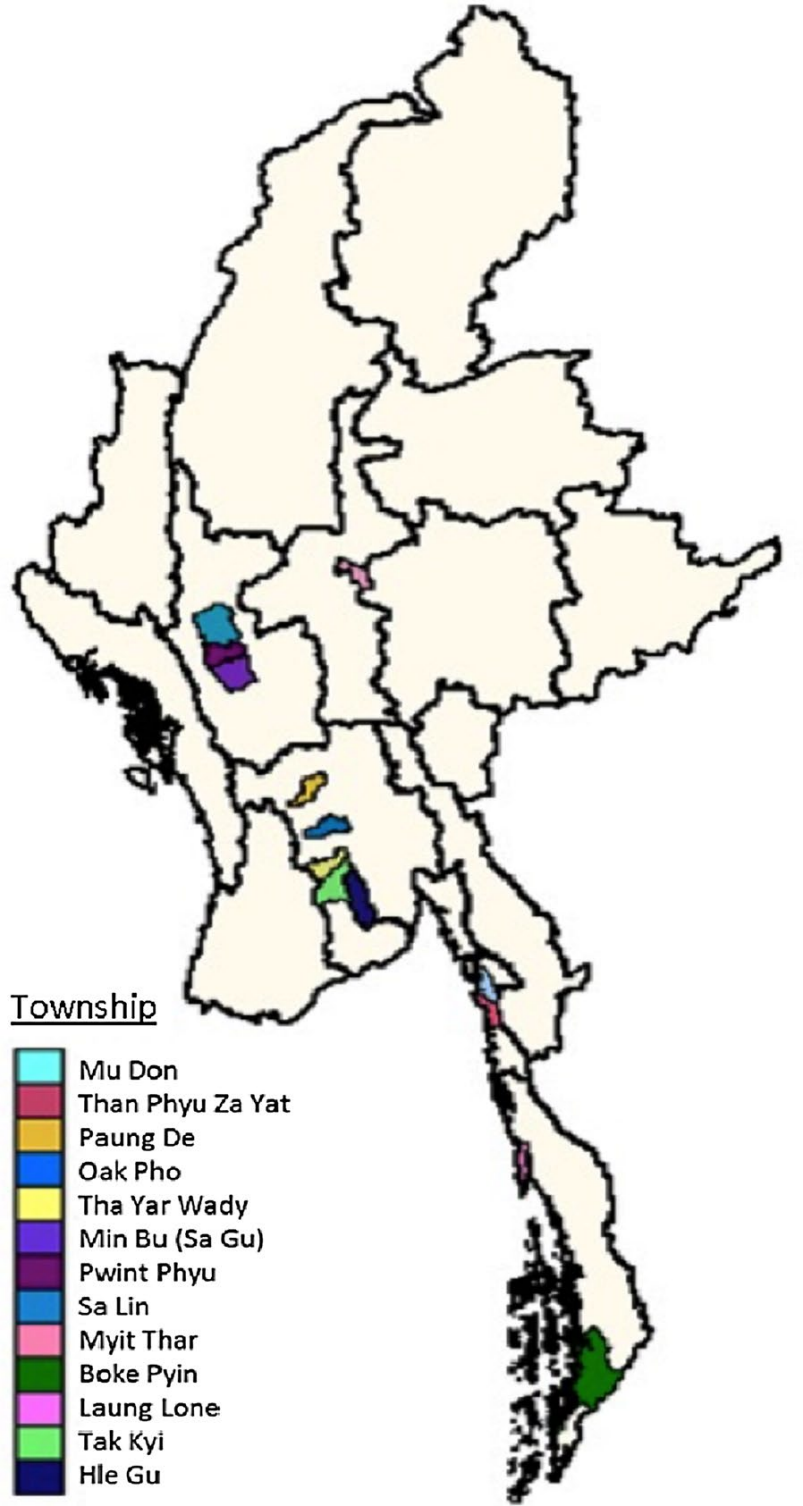
Map of the townships of Myanmar

The study was conducted between January 2011 and October 2012 in 13 Myanmar townships (see Fig. 1). The University of California, San Francisco (UCSF) Committee on Human Research approved this study on October 26, 2010.

### Provider selection and sampling

Starting in early 2011 PSI/M identified and recruited providers for the SPH programme in coordination with local health officials. Individuals in each rural community who had some prior health experience—most commonly as a traditional birth attendant or a government-trained community health worker—were recruited first. No inclusion/exclusion adjustments were made based on prior experience being private or public. If no individuals with experience existed, PSI/M identified potential recruits based on the advice of local officials.

SPH recruits had to complete two 3-day trainings, approximately 1 month apart. All individuals who completed the first of these two trainings were eligible for recruitment for this study. After the initial training, a researcher approached each provider and invited him/her to participate in the study. The researcher used a standard script for recruitment that explained the study goals, that participation was voluntary and unrelated to enrollment in the SPH programme, and that if the provider agreed, researchers would visit him/her at home three times over the coming year.

Providers were recruited for this study from each new cohort of trainees in the SPH programme between January 1, 2011 and August 30, 2011. A total of 257 recruits completed the first training, in 12 groups, averaging 20 trainees per group. Of this total, 251 agreed to participate in the study (97.7 %). Fifty-four providers (21 %) quit the SPH programme before the end of the study.

### Observed simulated patient

For this study, a hybrid methodology was applied incorporating attributes of simulated patient care and treatment observation and scoring. The methodology, called the Observed Simulated Patient (OSP), assesses the providers’ capacity to test, diagnose, and then treat paediatric malaria. OSP permits a unique standardized assessment of provider quality of care in the treatment of childhood illnesses, which cannot be assessed through mystery clients or other more common methods. The diagnostic and treatment actions required for proper care were based upon WHO protocols, and PSI/Myanmar guidelines (themselves based on WHO guidelines). A panel of experts was convened at the Myanmar Institute of Tropical Medicine to provide weights estimating the importance of each action to assuring proper care. These weights were adopted without further change. The OSP method is validated and described in detail elsewhere [26].

**Fig. 2:**
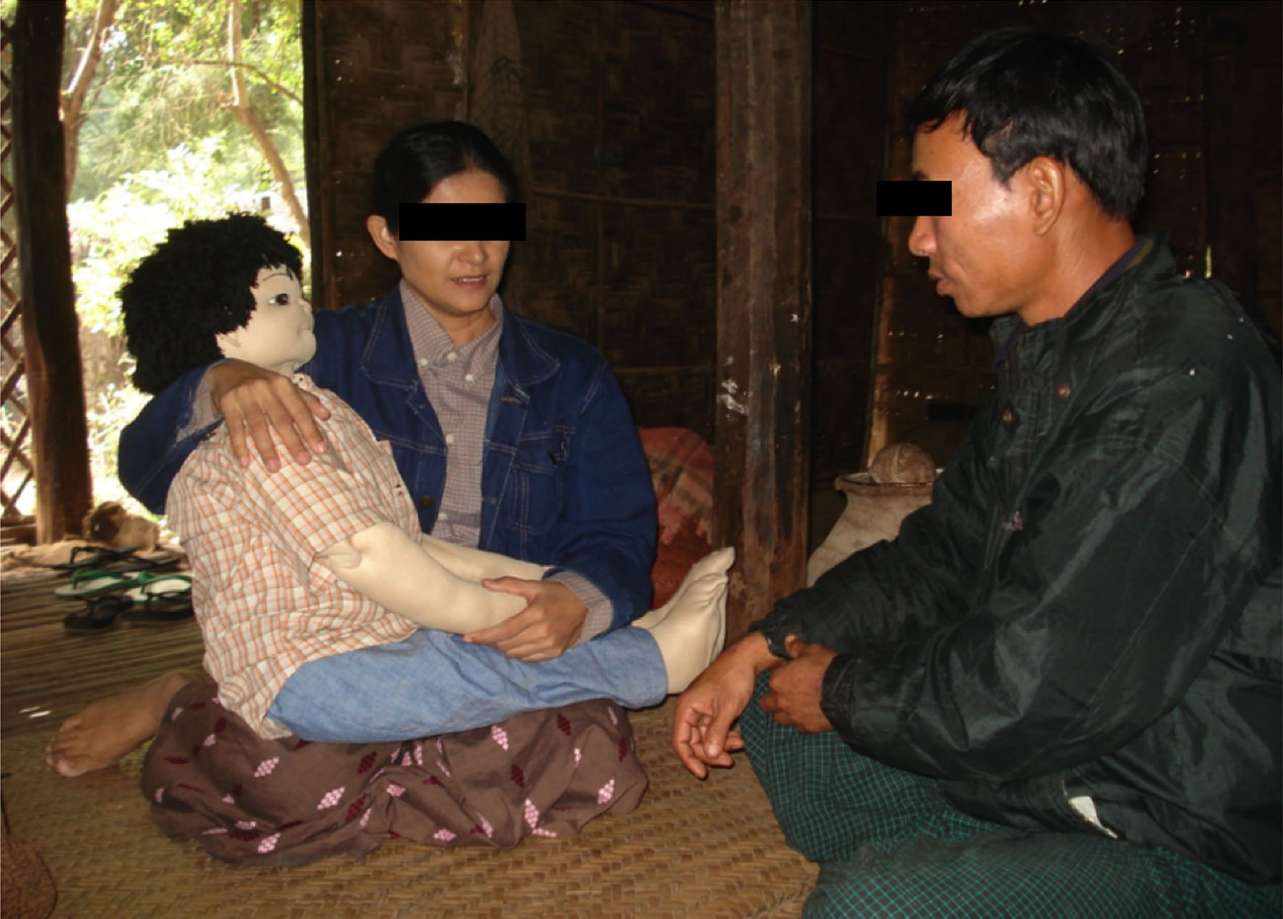
The observed simulated patient method in practice

The OSP method requires that a researcher trained as an actress play the role of a mother, using a doll of approximately the same size and weight of a 5-year-old male child (see Fig. 2). The researcher instructs the provider to examine the doll and propose treatment as if the doll were a real child. A second researcher scores the examination, which is measured against WHO-derived best practices for the diagnosis and treatment of non-severe paediatric malaria.

### Testing

The OSP baseline study was administered between the end of the first training (Day 3) and the beginning of the second training (Day 30) in the locations where SPH providers would see clients, usually in their home or in the yard in front of their home. Two trained research staff visited providers and introduced to the OSP methodology. After explaining the scenario to the provider and instructing him to diagnose and treat the OSP doll as if it were a real child, the assessment began.

**Fig. 3:**
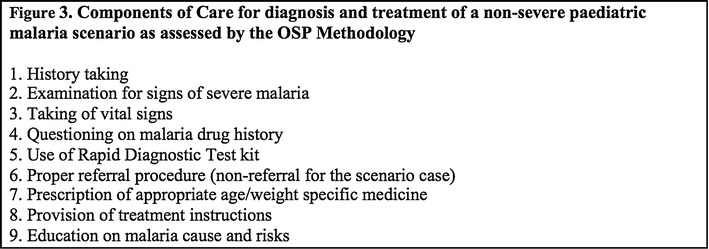
Components of care for diagnosis and treatment of a non-severe paediatric malaria scenario as assessed by the OSP methodology

Providers were evaluated on elements of care in nine areas (see Fig. 3). Binary scores assessed performance of specific activities, such as checking the child’s eyes for jaundice. During analysis, each activity performed was given a score weighted according to importance for assuring safe and effective health outcomes. For example, prescription of appropriate medication scored higher than patient history taking. Malaria experts set the weights prior to the start of the study. See Aung et al. [26] for a more complete description of the OSP methodology, scoring, and weighting system development, and measurements.

Providers were retested using the same OSP methodology at 6 and 12 months after their initial evaluation. For example, providers who completed the baseline OSP test in January 2011 were retested in July 2011 and then again in January 2012. The same scenario and scoring system were used in all three tests.

### Data entry and storage

A unique code was attached to each paper-scoring sheet along with the SPH provider name, location, researcher information and the date of the assessment. Data collection teams returned scoring sheets to PSI/M at the end of each week. Research staff entered unique provider identifiers and scores into SPSS on a rolling basis, but excluded provider names. The research staff then aggregated an average provider score after each round of testing with all SPH providers was complete. All baseline data were completed and entered by September 2011; all second round surveys were completed and entered by March 2012; and all third round surveys were completed and entered by September 2012.

### Analysis

All data analysis was conducted in SPSS 15. Bivariate and multivariate analyses were run with dummy variables indicating each survey round. Provider quality scores were measured against demographic characteristics (age, gender, education, and prior health training) in baseline and follow-up testing and used the same methods to look for correlations of changes in score. A separate analysis was conducted to compare the characteristics of the 54 providers who dropped out of the SPH programme with the 197 who remained throughout the study.

## Results

Of the 251 providers recruited to be in the study, there was an average of 21 recruits per township, with a minimum of seven from Hlegu/Taikgyiand a maximum of 34 from Tharyarwady (Table 1). Only two of the twelve townships retained all of their original recruited providers through Round 3 (12 months). Three additional townships lost only one provider and all of those losses came between Rounds 2 and 3. Overall, the retention rate of the study cohort was 88.9 % after 6 months and 78.5 % after 12 months, but retention was as low as 38.8 % (among providers recruited from Thanphyuzayet). The mean age of the SPH provider cohort was 31 years (range 24–39 years) and two-thirds were female. Over 75 % completed middle school and half of SPH providers had some prior health training.

**Table 1:** Demographic characteristics of SPH providers

Township	Number of providers			Age in years (mean) N = 197	Female gender (%) N = 197	Completed middle school (%) N = 197	Previous health experience (%) N = 197
	Before training	6 m after training	12 m after training				
Minbu	22	21	20	33	55.0	75.0	100.0
Myittha	14	14	14	39	64.3	71.4	42.9
Mudon	23	17	15	24	100.0	93.3	33.3
Paungde	29	27	25	36	48.0	72.0	92.0
Boat Pyin	13	13	12	31	91.7	58.3	75.0
Thanphyuzayet	18	14	7	31	85.7	100.0	42.9
PwintPhyu	12	12	11	32	63.6	72.7	27.3
Salin	24	24	23	34	52.2	65.2	65.2
Hlegu/Taikgyi	7	7	7	29	85.7	85.7	57.1
Tharyarwady	34	32	28	27	60.7	78.6	3.6
Oat Pho	24	18	16	29	81.3	93.8	37.5
Long Lon	31	24	19	26	68.4	84.2	36.8
Total	251	223	197	31	67.0	77.7	51.8

Before the SPH training, providers received low scores on their clinical quality for diagnosis and treatment of paediatric cases (Table 2, R1 mean = 11.97). Six months after training, mean scores for all nine “Components of Care” had improved, and the overall mean score had quadrupled (R2 mean = 48.02). At 12 months post-training, mean scores for eight of the nine components had decreased, although the decrease in the overall mean was small (R3 mean = 44.76).

**Table 2:** Performance scores on components of care (N = 197)

Clinical tasks	Possible score	Average score before training (baseline)	Average score at 6 m after training	Average score at 12 m after training
Medical history taking	5	2.28	2.84	2.68
Severe signs of malaria	13	1.4	2.9	2.16
Vital signs	8	2.56	3.53	2.74
Malaria drug history	5	0	0.07	0
Using RDT	30	1.6	22.92	22.56
Appropriate referral to a higher level facility	1	0.5	0.87	0.94
Prescribing proper drugs	10	0.61	4.72	4.44
Providing treatment instruction	17	0.39	6.52	5.97
Providing health education	11	2.63	3.65	3.27
Total score received	100	11.97	48.02	44.76

Between baseline and testing at 6 months, the use of rapid diagnostic test kits (RDTs) improved the most (71 %), followed by prescription of appropriate age/weight specific medicine (41 %) and provision of proper treatment instruction (36 %). In this same time frame, the taking of general medical history and malarial drug history improved the least (1.1 and 1.4 % increases, respectively).

The differences in means between providers’ scores across all three rounds of testing are presented in Table 3. Between rounds at R1 and R2, mean scores of seven of the nine “Components of Care” increased by a factor of 10 % or more. All nine components increased significantly from R1 to R2 (p < 0.05, p < 0.001 overall).

**Table 3:** Performance score differences between testing rounds (N = 197)

Testing round		(R2–R1)		(R3–R2)		(R3–R1)	
Clinical tasks	Possible score	Mean score difference (95 % CI)	p value	Mean score difference (95 % CI)	p value	Mean score difference (95 % CI)	p value
Medical history taking	5	0.56 (0.34, 0.76)	0.000	−0.16 (−0.35, 0.03)	0.091	0.39 (0.19, 0.59)	0.000
Severe signs of malaria	13	1.50 (1.06, 1.93)	0.000	−0.74 (−1.13, −0.35)	0.000	0.76 (0.36, 1.15)	0.000
Vital signs	8	0.97 (0.47, 1.48)	0.000	−0.79 (−1.18, −0.4)	0.000	0.18 (−0.29, 0.65)	0.443
Malaria drug history	5	0.07 (0.0, 0.14)	0.039	−0.07 (−0.14, 0.0)	0.039	NA	
Using RDT	30	21.32 (19.63, 23.01)	0.000	−0.36 (−2.04, 1.33)	0.678	20.96 (19.22, 22.71)	0.000
Appropriate referral to a higher level facility	1	0.37 (0.28, 0.45)	0.000	0.06 (0.01, 0.11)	0.018	0.43 (0.35, 0.51)	0.000
Prescribing proper drugs	10	4.11 (3.46, 4.77)	0.000	−0.28 (−0.87, 0.31)	0.349	3.83 (3.18, 4.49)	0.000
Providing treatment instruction	17	6.13 (5.34, 6.92)	0.000	−0.54 (−1.31, 0.23)	0.165	5.59 (4.94, 6.23)	0.000
Providing health education	11	1.02 (0.53, 1.5)	0.000	−0.38 (−0.82, 0.07)	0.098	0.64 (0.2, 1.08)	0.005
Total scores received	100	36.05 (33.1, 38.99)	0.000	−3.26 (−5.74, −0.78)	0.010	32.79 (29.96, 35.62)	0.000

A significant decrease was reflected in the total quality-of-care score between R2 and R3 (p < 0.01). Four mean scores exhibited significant decreases between R2 and R3: severe signs of malaria, vital signs, malaria drug history, and referral (p < 0.05). These declines, although significant, were small and for all but two components SPH quality scores in R3 remained higher than at baseline (p < 0.01).

The bivariate analysis presented several significant associations between Round 2 scores and the four demographic characteristics (Table 4). Older providers (>28 years) scored significantly higher than younger providers in taking of vital signs (p = 0.047) and in their overall quality score (p = 0.044). Female providers were better than their male counterparts at taking medical history (p = 0.016), administering malarial drug history (p = 0.039), and providing health education (p = 0.020). Completing formal education at or above middle school level was correlated with better scores for administering malarial drug history (p = 0.039) and delivering health education (p = 0.046). Previous health experience correlated with higher scores for evaluating indications of severe malaria (p = 0.025) and taking vital signs (p = 0.022).

**Table 4:** Bivariate association between the second round quality assessment scores and demographic characteristics

t’ test	Age (>28 years)		Female gender		Completed middle school		Previous health experience	
	Mean score difference (95 % CI)	p value	Mean score difference (95 % CI)	p value	Mean score difference (95 % CI)	p value	Mean score difference (95 % CI)	p value
Medical history taking	−0.14 (−0.49, 0.2)	0.410	0.45 (0.09, 0.81)	0.016	0.29 (−0.12, 0.7)	0.168	0.19 (−0.15, 0.54)	0.265
Severe signs of malaria	0.75 (−0.12, 1.62)	0.090	0.38 (−0.55, 1.3)	0.424	0.31 (−0.74, 1.36)	0.562	0.98 (0.13, 1.84)	0.025
Vital signs	0.77 (0.01, 1.52)	0.047	0.22 (−0.64, 1.09)	0.612	0.80 (−0.11, 1.71)	0.084	0.89 (0.13, 1.64)	0.022
Malaria drug history	0.10 (−0.03, 0.24)	0.137	0.11 (0.01, 0.21)	0.039	0.09 (0.0, 0.18)	0.039	0.1 (−0.03, 0.23)	0.147
Using RDT	3.13 (−0.1, 6.35)	0.057	−2.76 (−6.03, 0.51)	0.097	1.71 (−2.19, 5.61)	0.388	0.45 (−2.8, 3.71)	0.784
Appropriate referral to a higher level facility	−0.02 (−0.12, 0.07)	0.656	−0.01 (−0.11, 0.09)	0.797	−0.02 (−0.14, 0.09)	0.685	0.07 (−0.03, 0.17)	0.150
Prescribing proper drugs	0.35 (−0.9, 1.61)	0.581	0.62 (−0.72, 1.95)	0.364	0.52 (−0.92, 2.9)	0.499	0.58 (−0.68, 1.84)	0.365
Providing treatment instruction	0.96 (−0.63, 2.55)	0.234	0.06 (−1.63, 1.75)	0.943	0.99 (−0.92, 2.9)	0.308	−0.93 (−2.53, 0.67)	0.251
Providing health education	0.27 (−0.54, 1.08)	0.512	1.02 (0.16, 1.87)	0.020	0.98 (0.02, 1.95)	0.046	0.38 (−0.44, 1.2)	0.359
Total score	6.16 (0.16, 12.17)	0.044	0.07 (−6.38, 6.52)	0.983	5.67 (−1.57, 12.91)	0.124	2.7 (−3.35, 8.77)	0.378

Multivariate analysis did not show any significant association between SPH provider quality scores and demographic characteristics. Between baseline and the second round of testing 28 providers (11 %) dropped out of the SPH programme (Table 5). Between the second and third rounds of testing, another 26 providers (12 %) left the SPH programme. Providers who remained in the SPH programme were 2.5 times more likely than those who quit to be older (>28 years, p = 0.005) and three times more likely to have had some prior health training (p = 0.001).

**Table 5:** Comparative demographic characteristics of participants versus dropouts

	Participated in all 3 rounds of testing (N = 197) (%)	Dropouts (N = 54) (%)	Odds ratio	p value	95 % confidence interval	
					Lower	Upper
Age (>28 years)	49.7	27.8	2.57	0.005	1.33	4.97
Female gender	67	72.2	0.781	0.513	0.402	1.519
Completed middle school	77.7	81.5	0.79	0.709	0.368	1.697
Previous health experience	51.8	25.9	3.068	0.001	1.57	5.993

## Discussion

The PSI/M training on malaria prevention and care improved the quality of care delivered by SPH providers on all nine Components of Care assessed by the OSP methodology. Scores were significantly better both at 6 and at 12 months after the training than at baseline. As a result of the PSI/M SPH programme, 197 providers were added to the rural health workforce with a greatly improved capacity to test, diagnose, and treat malaria.

The largest change in score was the use of RDTs. Since RDT use was the most heavily weighted of the nine Components of Care—comprising 30 % of the overall score—the 71 % score increase between R1 and R2 was particularly promising. Almost two thirds of the total score increase from R1 to R2 was a direct result of the increased use of RDT (21.32 out of 36.05 points). Also of note were the 41 % score increase in prescription of appropriate age/weight specific medicine and 36 % score increase in provision of proper treatment instruction. While the other six Components of Care are important, these three skills—RDT, accurate prescription, and proper instruction—are some of the most essential. Increases in scores denote a particularly favorable uptake of critical skills.

Each of the four demographic characteristics had significant effects on individual Components of Care scores, but the only attribute that affected overall score was being over 28 years of age. This finding suggests that older providers may be better or more motivated providers, and should be targeted in future recruitment efforts. Individuals over 28 years were also less likely to dropout of the SPH programme. Because provider attrition is a very real concern in the rural Myanmar health system, recruiting older providers may also improve retention rates.

Providers with previous healthcare experience scored better on evaluating severe signs of malaria and taking vital signs than their counterparts without prior healthcare experience; however, no significant difference was found between the two groups in terms of overall quality of care, indicated by their total score. Providers with previous healthcare experience were, however, significantly less likely to dropout of the programme, than those without previous experience, which suggests that prior interest in healthcare represents a maintained interest. While programmes like SPH can recruit individuals without previous health experience and they will score just as well as those with prior healthcare experience, those with healthcare experience are more likely to stay involved, again addressing the real concern of attrition.

Almost one quarter of all recruited providers left the SPH programme at some point during the 12 months following training. The scores of those who left the programme to those who stayed were not compared. Additional analysis is warranted to determine whether providers with higher scores were more likely to continue with the programme.

While quality scores remained higher after 12 months than at baseline, scores declined between testing at 6 and 12 months. This may be due to a lack of intermediate “refresher” courses offered during the yearlong study. Studies have shown regular, continuing education courses for community health workers maintain knowledge, accuracy, and quality of practice [27–29]. Perhaps instead of a one-off training, SPH should conduct routine refresher courses that would allow for reinforcement of previous training and provide opportunities to update and improve quality of care. In addition, more regular engagement has been exhibited to decrease rates of health worker attrition [30, 31].

Although the scores received by SPH providers at Round 2 were four-times better than at baseline, these scores still remained less than 50 % of the maximum achievable score and well below the scores of the 20 experienced physicians who were assessed using the same methodology. Too often care for the poor has been poor care, especially in rural areas.

There are several limitations to this study. The OSP scoring for malaria, based on WHO and Myanmar MOH guidelines, only included positive scenarios. There is no system for giving negative points to providers who offered potentially harmful advice. More importantly, the scoring system does not have an established cut-off for acceptably safe care. Evaluations were conducted previously with long-established medical doctors in Myanmar to provide a baseline score of approximately 70/100 [12]. While the quality of care delivered by SPH providers is much higher after training in contrast to the other care options available in study communities, it is difficult to say that the quality of care given by SPH providers is ‘sufficient’ from an objective measurement standpoint. While the list of activities needed to provide proper diagnosis and care is based upon WHO standards, the weights given to each activity were determined by local malariologists and infectious disease experts and therefore may not match the weights which may be given by clinicians in other fields or other countries.

## Conclusions

Informed recruitment, consistent and frequent training, and setting and maintaining an accreditation standard comprise a framework of methods that can improve quality of care in similar rural settings. Quality begins with recruitment and retention. This study showed that providers that were over 28 years old and had previous health training were more likely to score higher and to remain in the programme. Once good providers are recruited and retained, assuring their capacity to provide quality care is required. This study showed that a one-time training yielded a fourfold increase in the provision of necessary components of care to treat paediatric malaria. However, this score was still less than half of the possible achievable score, and 40 % less than the average score of private physicians tested in a prior study. Assuring the best method to sustain improvements over time should be a priority for programmes providing malaria treatment.

Finally, a standard must be set for the level of quality that is acceptable. “Acceptability” must be addressed first, and then followed by “achievability” as the expectations for standards of quality of care continue to rise and be met. Future research is needed to determine the probability of negative health effects with, and without, treatment at different levels of quality. Progress against these aspects of care provision and assurance of quality can greatly improve malaria treatment in Myanmar as well as other endemic countries, and have meaningful health impact.
